# Ultrasonication effects on thermal and rheological properties of carbon nanotube suspensions

**DOI:** 10.1186/1556-276X-7-127

**Published:** 2012-02-14

**Authors:** Binglu Ruan, Anthony M Jacobi

**Affiliations:** 1 Mechanical Science and Engineering, University of Illinois, Urbana, IL, 61801-2906, USA; 2 Department of Thermal Engineering, Tsinghua University, Beijing, 100084, China

## Abstract

The preparation of nanofluids is very important to their thermophysical properties. Nanofluids with the same nanoparticles and base fluids can behave differently due to different nanofluid preparation methods. The agglomerate sizes in nanofluids can significantly impact the thermal conductivity and viscosity of nanofluids and lead to a different heat transfer performance. Ultrasonication is a common way to break up agglomerates and promote dispersion of nanoparticles into base fluids. However, research reports of sonication effects on nanofluid properties are limited in the open literature. In this work, sonication effects on thermal conductivity and viscosity of carbon nanotubes (0.5 wt%) in an ethylene glycol-based nanofluid are investigated. The corresponding effects on the agglomerate sizes and the carbon nanotube lengths are observed. It is found that with an increased sonication time/energy, the thermal conductivity of the nanofluids increases nonlinearly, with the maximum enhancement of 23% at sonication time of 1,355 min. However, the viscosity of nanofluids increases to the maximum at sonication time of 40 min, then decreases, finally approaching the viscosity of the pure base fluid at a sonication time of 1,355 min. It is also observed that the sonication process not only reduces the agglomerate sizes but also decreases the length of carbon nanotubes. Over the current experimental range, the reduction in agglomerate size is more significant than the reduction of the carbon nanotube length. Hence, the maximum thermal conductivity enhancement and minimum viscosity increase are obtained using a lengthy sonication, which may have implications on application.

## Introduction

Thermal conductivity and viscosity of a heat transfer fluid play an important role in efficiency improvement of thermal equipment and systems as: air-conditioning and refrigeration, transportation, electronic cooling, heating and ventilating, etc. Researchers have found many ways to enhance the thermal conductivity of a heat transfer fluid, including suspending solid particles into the fluid. However, micrometer or millimeter-sized particles suspended in the fluid usually settle and can cause corrosion and abrasion to the components and systems. Recently, developments in nanotechnology made nanometer-sized particles available. In 1995, Choi and Eastman [1] firstly introduced the nanometer-sized particles (nanoparticles) into heat transfer fluids and coined the term 'nanofluid.'

Many researchers found that dispersing a small amount of nanoparticles into a heat transfer fluid can enhance its thermal conductivity dramatically, and the enhancement could be beyond that expected from the conventional mixing theory, such as Maxwell theory [2] and Hamilton-Crosser theory [3]. Eastman et al. [4] observed a 40% thermal conductivity by dispersing 0.3 vol% copper nanoparticles into ethylene glycol. Choi et al. [5] investigated the thermal conductivity of carbon nanotube-oil suspensions and obtained a 150% enhancement for the nanofluid with a concentration of 1.0%. Das et al. [6] explored temperature effects on the thermal conductivity enhancement of nanofluids and found that dispersion of nanoparticles into the fluid can significantly enhance its thermal conductivity, and a larger enhancement can be observed at an elevated temperature. For the viscosity of nanofluids, some researchers found no significant change compared to the base fluid [7]. However, other researchers also noticed a remarkable increase in viscosity for the fluid with nanoparticles. Murshed et al. [8] observed 60% and 80% viscosity increases for Al_2_O_3_-water and TiO_2_-water nanofluids with concentrations of 3 vol%, which is more significant than the predictions of Krieger-Dougherty's [9] and Nielsen's models [10]. The viscosity of Al_2_O_3_-water nanofluids prepared by Pak and Cho [11] was almost three times higher than that of pure water. Ruan and Jacobi [12] obtained no significant falling-film convective heat transfer enhancement and attributed it to a 12.5% viscosity increase and 4.6% thermal conductivity enhancement that they had observed for Al_2_O_3_-water nanofluids with concentrations of up to 2 vol%. Wang et al. [13] measured the viscosity of the same kind of nanofluids with different dispersion techniques, and stated that the nanofluid could have a lower viscosity if the particles were better dispersed. Hence, the nanofluid preparation could be a key to determine the performance of the nanofluids.

Generally, there are two methods to disperse the nanoparticles into base fluids: a so-called one-step method and a two-step method [1]. The two-step method is widely used since a larger amount of nanofluid can be prepared at one time. Moreover, the two-step method is suitable for nonmetallic nanoparticles and base fluids with high vapor pressures. When preparing nanofluids by the two-step method, nanoparticles are dispersed into the base fluid, and then the suspension is treated by a mechanical method to reduce aggregation in the suspension. Ultrasonication is probably the most widely used and most effective mechanical technique for this purpose. Many researches use a bath or tip sonicator to treat their nanofluid samples [14-16]; however, very limited work of ultrasonication effects on the nanofluid preparation is reported in the open literature. Amrollahi et al. [17] investigated the ultrasonication time effects on sediment and the thermal conductivity of the carbon nanotube-ethylene glycol nanofluids and found that thermal conductivity of the nanofluids increased with sonication time. Yang et al. [18] explored the sonication energy/time impact on thermal conductivity of nanotube-oil suspensions and observed a decreased thermal conductivity with an increasing sonication energy/time. They also investigated the sonication energy effects on steady-shear viscosity of nanotube-oil suspensions and found that the viscosity decreased with increased sonication energy.

In this study, the ultrasonication effects on thermal conductivity and viscosity of ethylene glycol-based multiwall carbon nanotube [MWCNT] nanofluids are explored. (Sun Innovations Inc., Fremont, CA, USA) Optical microscope, scanning electron microscope [SEM] and transmission electron microscope [TEM] images of samples, subjected to different sonication times, are used to explore the sonication effects on the size of agglomerates and the length of the nanotubes, which are significant factors affecting the thermal conductivity and the viscosity of the nanofluids. The results are compared to the available literature and possible explanations for the observed behavior are offered.

## Experimental details

### Materials

**Figure 1:**
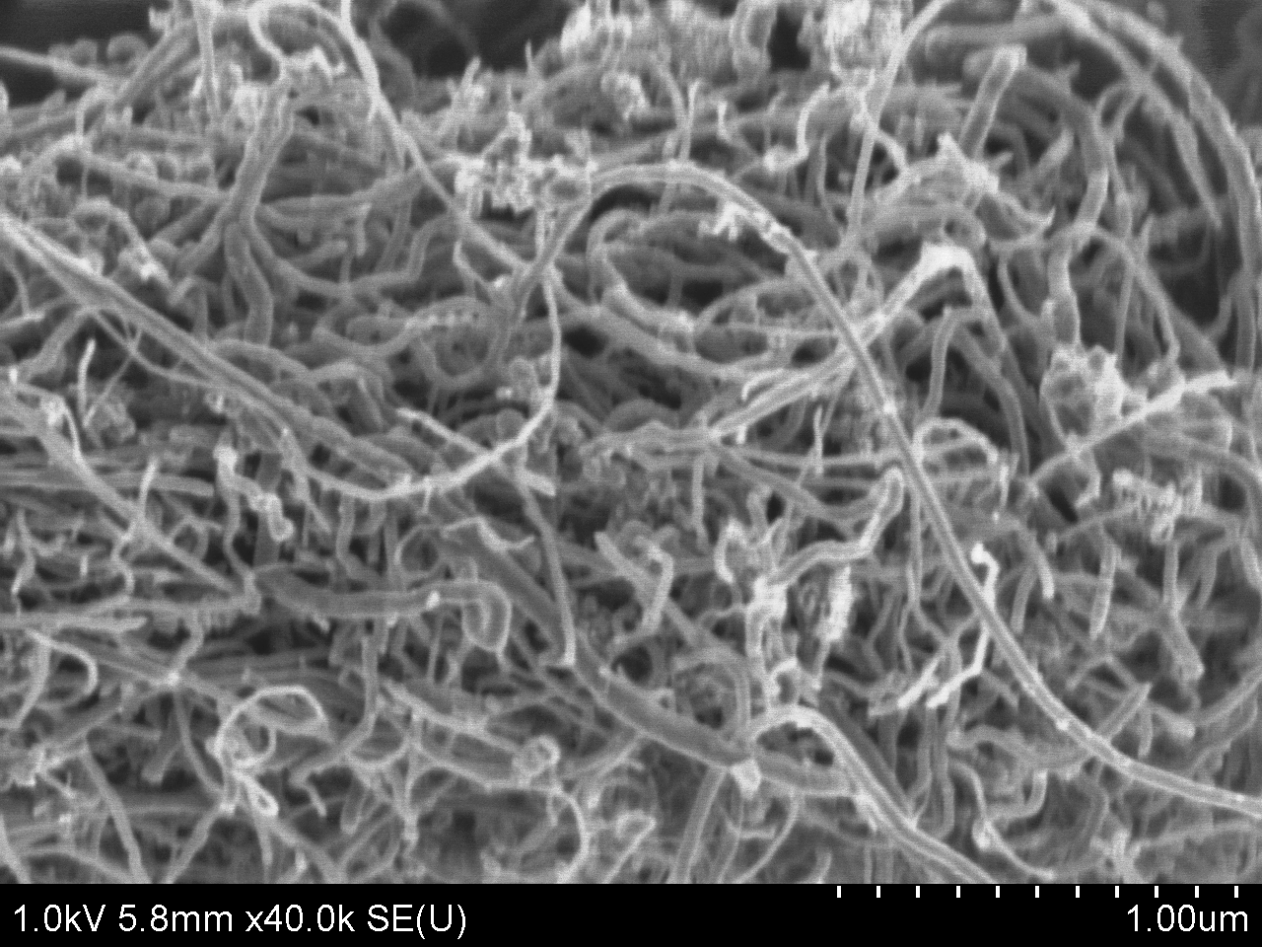
**SEM image of the MWCNTs as received**.

The multiwall carbon nanotubes have nominal outer diameters of 10 to 30 nm, inner diameters of 5 to 10 nm and lengths of 10 to 30 μm. The MWCNTs were manufactured by chemical vapor deposition. An SEM micrograph of the MWCNTs as received is provided in Figure 1.

### Nanofluid preparation

**Figure 2:**
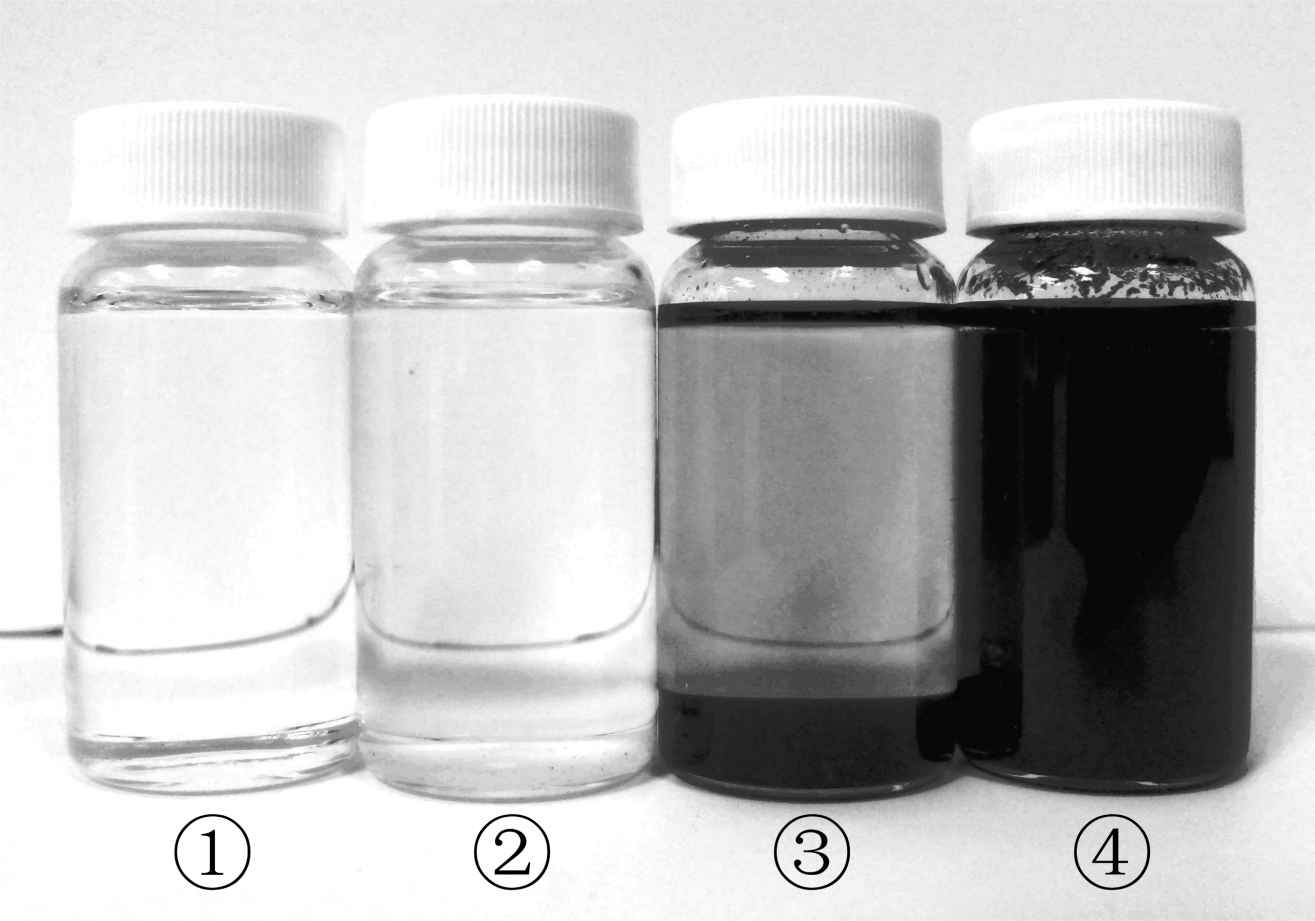
**Images of ethylene glycol-based MWCNT nanofluids, after sitting still for 1 week in the laboratory**. From left to right: sample 1, pure ethylene glycol; sample 2, ethylene glycol with 0.25 wt% gum arabic; sample 3, ethylene glycol with 0.25 wt% gum arabic and 0.5 wt% MWCNT, no sonication; sample 4, ethylene glycol with 0.25 wt% gum arabic and 0.5 wt% MWCNT, sonicated continuously for 120 min.

Considering that the surface of the carbon nanotube is hydrophobic and ethylene glycol is a polar liquid, gum arabic was employed as a dispersant in order to better disperse the carbon nanotubes in the ethylene glycol. When preparing the nanofluid, the gum arabic at a concentration of 0.25 wt% was first dispersed into the ethylene glycol in a 500-ml glass breaker, which was placed on a stirrer with a stir bar rotating inside the fluid; after the gum arabic was fully dissolved into the ethylene glycol, 0.5 wt% MWCNTs were dispersed into the fluid. A tip ultrasonicator was used to treat the fluid at settings of 150 W both continuously and in pulse 20 mode (0.8 s on and 3.2 s off) at 20 kHz. The ultrasonicator has a timer to set the desired sonication time. Based on the known volume of the test liquid (500 ml), the specific sonication energy per minute can be calculated as 1.8 × 10^4 ^kJ/m^3^. Hence, the sonication energy can be obtained as the specific sonication energy per minute multiplied by the sonication time. For the pulse mode, the sonication energy was calculated as the energy at the continuous mode multiplied by the percentage of the 'on' time (e.g., 20% for the pulse 20 mode). As an initial screening of the effectiveness of sonication, a sonicated nanofluid and an unsonicated specimen were placed still in the lab for more than 1 week to assess settling. A photograph of the specimens is shown in Figure 2, where it is shown that a sonicated specimen manifested no significant settling, but an unsonicated nanofluid had a thick layer of sediment (Figure 2, samples 3 and 4).

### Agglomerate and particle size observation

The size of agglomerates in the nanofluids was examined using an optical microscope. At least four images at different locations were recorded for each nanofluid sample to ensure the accuracy of the test. The images recorded with the microscope were analyzed by standard image processing methods, and the average sizes of agglomerates and their uncertainties were determined. The lengths of MWCNTs in suspensions at different sonication times were determined using TEM. Since the quantity of MWCNTs in each TEM sample was limited due to the method of TEM sample preparation, at least five TEM samples were prepared for each nanofluid sample and four images for each TEM sample were recorded. The TEM images were also analyzed using standard image processing methods, and the average lengths of MWCNTs and their uncertainties were obtained. The standard image processing methods included pixelization, threshold definition, binary conversion, and geometry analysis.

### Thermal conductivity and viscosity measurements

Thermal conductivity, *k*, of the suspensions was measured using a manufacturer-calibrated KD2-pro thermal property meter (Decagon Devices, Pullman, WA, USA) at the room temperature (20°C). The instrument is based on the hot-wire method. The probe (60 mm in length and 1.3 mm in diameter) of the KD2-pro thermal property meter integrates a heating element and a thermometer. Both the heat output and the temperature rise of the probe were recorded and sent to a microprocessor to calculate thermal properties of the test fluid. The uncertainty of the KD2-pro for thermal conductivity measurement as indicated by the manufacturer is ± 5%; however, by repeating measurements for the same fluid for more than 50 times, it was found that the repeatability, which is relevant in determining changes in thermal conductivity was ± 3%. Since the MWCNT-ethylene glycol nanofluid behaved as a non-Newtonian fluid, the viscosity, *μ*, was measured using a stress-controlled rotational rheometer at 20°C. The system had a torque range of 0.5 μNm-100 mNm, and a resolution of 1 nNm. A 4°/40 mm cone-plate measurement unit was used. The test sample was placed on the 20°C thermostat plate with the temperature maximum deviation of ± 0.01°C, after well shaken in the test tube. Once the temperature of the sample reached a steady state, the measurements were started. As the shear stress was applied, the rotational speed of the cone and cone dimensions gave the shear rate. The start and end shear stresses were 0.02 and 5.5 Pa, respectively, and the shear rate range was 10 to approximately 100 s^-1^. The apparent viscosity was calculated by the power law model. The measurements were repeated for five times for each test sample to ensure the accuracy, and the maximum deviation was found to be less than 5%.

## Results and discussion

### Thermal conductivity

**Figure 3:**
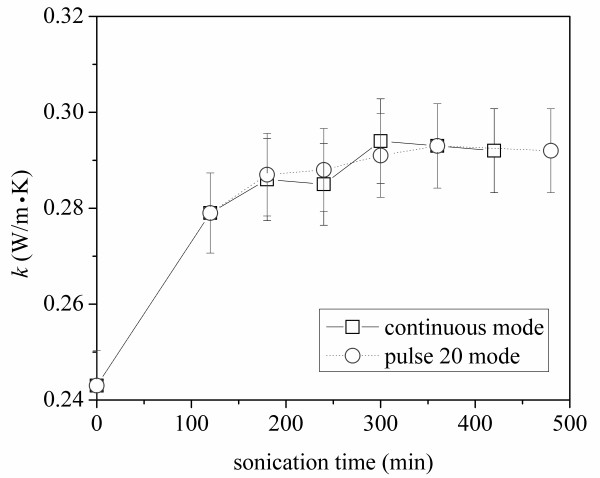
**Sonication time effect on thermal conductivity of MWCNT-ethylene glycol suspensions**. This is a comparison of continuous mode and pulse mode.

In preliminary experiments, both pulse mode and continuous mode sonications were used to treat otherwise identical samples and the thermal conductivity of the treated fluids samples was measured and compared. During sonication at 20 kHz, bubbles are created and collapsed, and the resulting shock from this cavitation process breaks up nanotube agglomerates. However, the process also generates heat and the nanofluid temperature rises, especially in continuous mode sonication. In order to mitigate evaporation of the base fluid during sonication, a cooling system was employed during the continuous mode sonication, maintaining a sample temperature at about 20°C. The thermal conductivity data for the two identical samples with different sonication modes are provided in Figure 3, where each reported measurement is the average of five readings, and the data have a two-σ precision limit of ± 3%. From the figure, it can be seen that the sonication mode had very little impact on the thermal conductivity; the differences are within the precision limit. For convenience, pulse mode sonication was adopted as the standard procedure for later experiments.

**Figure 4:**
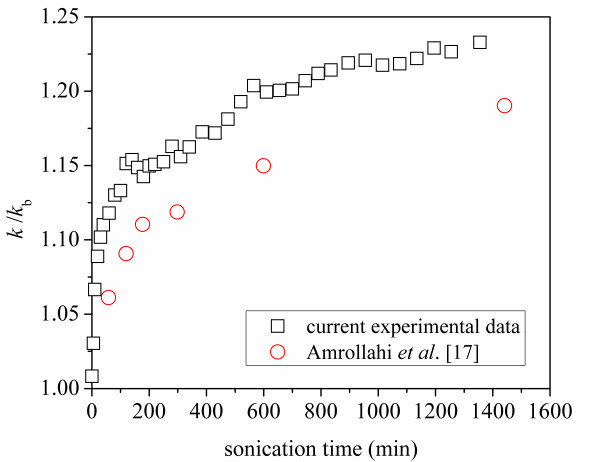
**Thermal conductivity ratio variation with sonication time for 0.5 wt% MWCNT-ethylene glycol suspensions**. This is a comparison between experimental data with dispersant and data from Amrollahi et al. [17] without dispersant.

**Figure 5:**
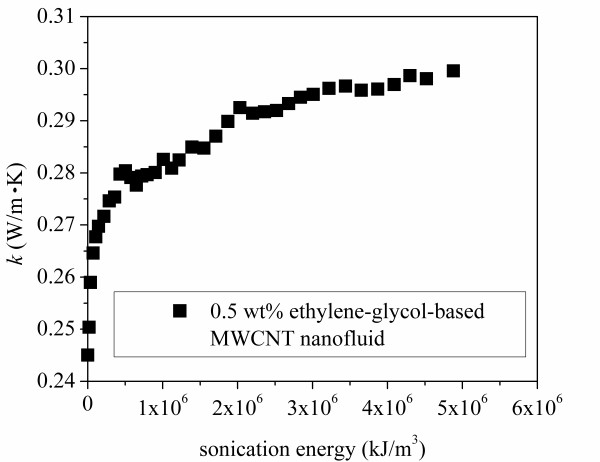
**Thermal conductivity variation with sonication energy for 0.5 wt% MWCNT-ethylene glycol suspensions**.

The ratio of the nanofluid thermal conductivity to that of the base fluid, *k*/*k*_b_, is plotted against sonication time in Figure 4. The thermal conductivity ratio, *k*/*k*_b_, of ethylene glycol with 0.25 wt% gum arabic only was measured to be 1.02. It can be seen in Figure 4 that the thermal conductivity of the nanofluid always increased with the sonication time. The increase in thermal conductivity was more significant during the first 160 min. After around 22 h of sonication, the thermal conductivity reached a value 23% larger than that of the base fluid, and the data suggest an asymptotic value not much larger than that achieved after 22 h. By comparing our data to those of Amrollahi et al. [17], it can be seen that the thermal conductivity increase in the current experiments is around 5% larger than that found by Amrollahi et al. [17] for 0.5 wt% MWCNT-ethylene glycol suspensions. The larger increase may be caused by the dispersant used in the current experiment. Amrollahi et al. [17] dispersed carbon nanotubes directly into ethylene glycol without any dispersant. Moreover, it was found in our previous work [19] that an addition of a small amount of gum arabic itself in pure ethylene glycol slightly increases its thermal conductivity. However, the thermal conductivity of MWCNT-ethylene glycol suspensions was found to be insensitive to gum arabic concentrations (from 0.1% to 3%). The variation of the thermal conductivity of the nanofluid with the ultrasonication input energy per unit volume is shown in Figure 5. The energy input per unit volume is 1.8 × 10^4 ^kJ/m^3 ^for a sonication time of 5 min at pulse 20 mode.

### Viscosity

**Figure 6:**
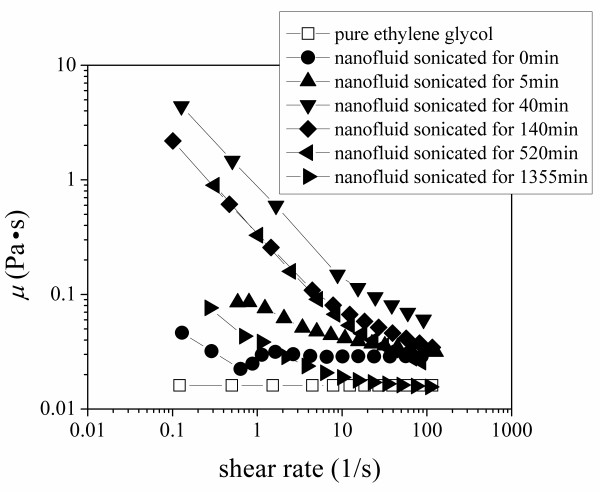
**Rheological behavior of MWCNT-ethylene-glycol nanofluid at different sonication time**.

**Figure 7:**
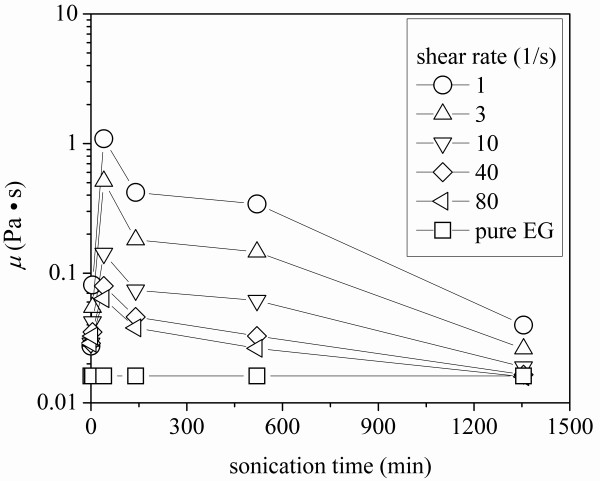
**Viscosity of MWCNT-ethylene-glycol nanofluid variation with sonication time at different shear rates**.

**Figure 8:**
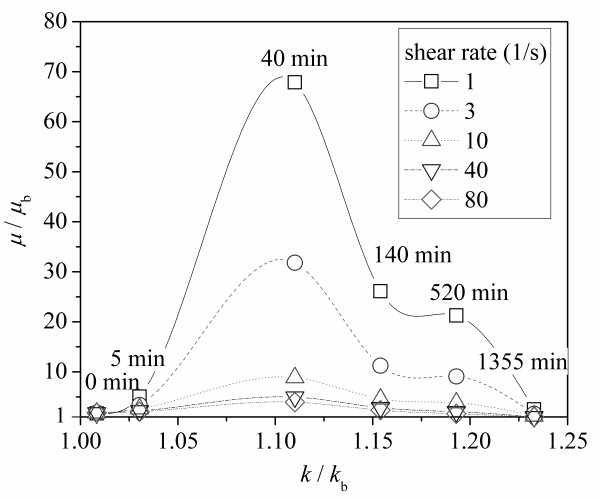
**Viscosity increase of MWCNT-ethylene-glycol nanofluid variation with thermal conductivity enhancement at different shear rates**.

The rheological behavior of MWCNT-ethylene glycol nanofluids after different sonication times is shown in Figure 6. The viscosity of the pure ethylene glycol was recorded before the nanofluid viscosity measurements and compared to the values from literature to verify the system accuracy. The result shows no dynamic viscosity change with the shear rate for pure ethylene glycol; however, the results for carbon nanotube suspensions displayed a shear thinning behavior, which was also observed by Yang et al [18]. When comparing the rheological behavior of samples subjected to different sonication times, it is found that the nanofluid with the sonication time of 40 min has the highest viscosity, and its viscosity decreased dramatically (from 4.4 to 0.06 Pa.s) with an increase in shear rate (from 0.1 to 100/s). However, the viscosity of the sample with the sonication time of 1,355 min displays a more flat viscosity variation with an increasing shear rate; moreover, at higher shear rates, its viscosity approached that of the base fluid. This behavior can be observed more clearly in Figure 7, where the viscosity of the test sample is plotted against the sonication time at different shear rates. It can be seen from Figure 7 that the viscosity at low shear rates is larger than that at higher shear rates at a fixed sonication time. It is interesting that at a fixed shear rate, the viscosity of the nanofluid first increased then decreased with an increase in sonication time. When the nanofluid is sonicated for around 40 min, the viscosity sharply increased to the maximum, and with further sonication, the viscosity of the nanofluid decreased gradually until it approached the viscosity of pure ethylene glycol for long sonication times. The nanofluid viscosity increase, *μ*/*μ*_b_, is plotted against the thermal conductivity ratio, *k*/*k*_b_, at different shear rates in Figure 8. It is clear in Figure 8 that the viscosity increased firstly and decreased, with increased sonication time and thermal conductivity. Finally, the largest thermal conductivity and lowest viscosity were obtained by a long sonication time for ethylene glycol-based MWCNT nanofluids. This finding may be very important for the heat transfer applications of nanofluids.

### Agglomerate size

**Figure 9:**
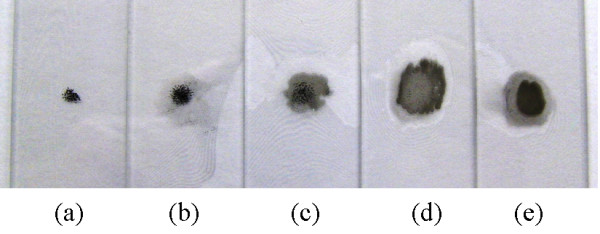
**MWCNT-ethylene-glycol nanofluid samples between glass slides for different sonication time**. From left to right: (**a**) 5 min, (**b**) 40 min, (**c**) 140 min, (**d**) 520 min, (**e**) 1,355 min.

**Figure 10:**
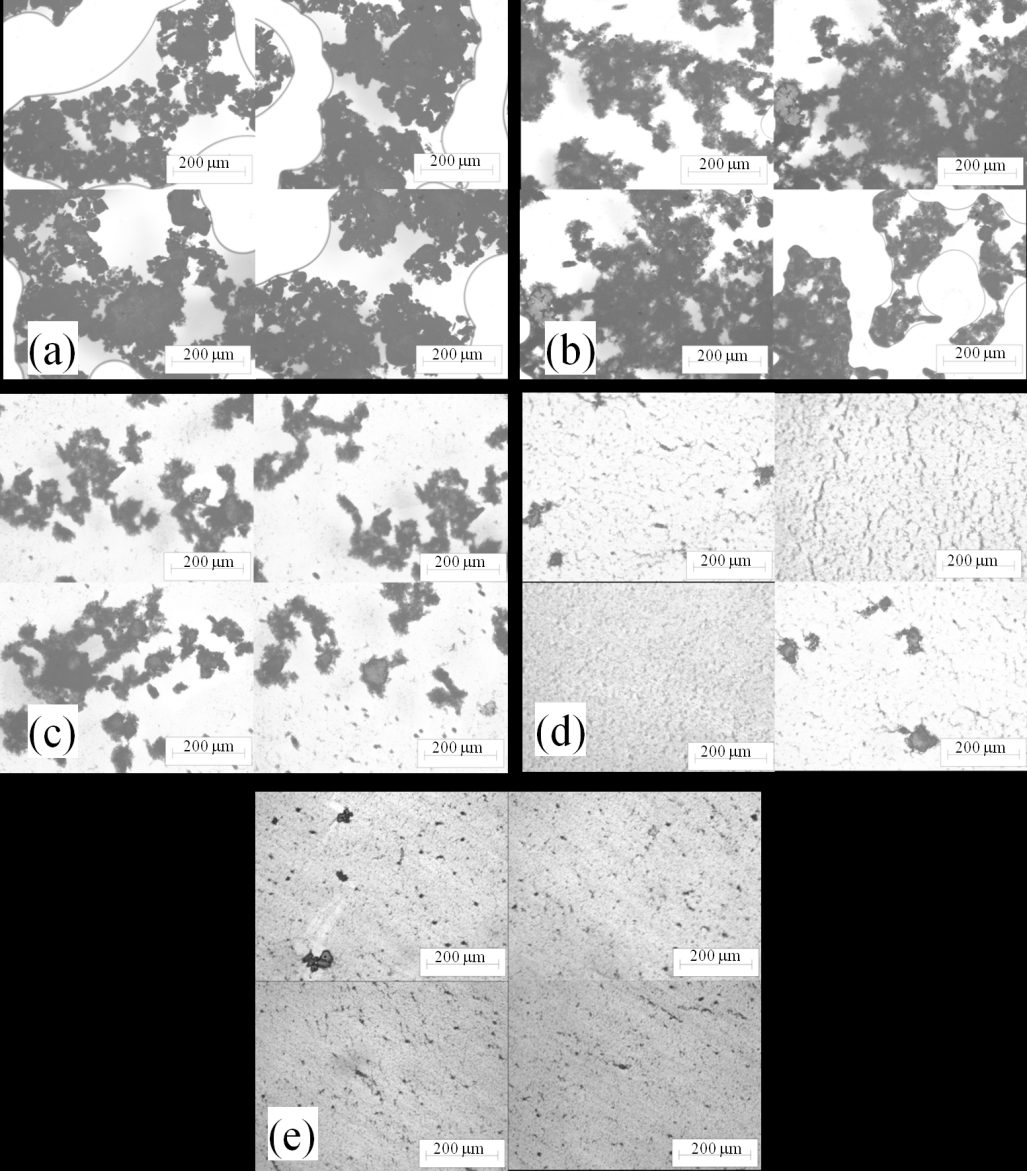
**Micrographs of MWCNT-ethylene glycol nanofluids subjected to different sonication times**. (**a**) 5 min, (**b**) 40 min, (**c**) 140 min, (**d**) 520 min, and (**e**) 1,355 min.

**Figure 11:**
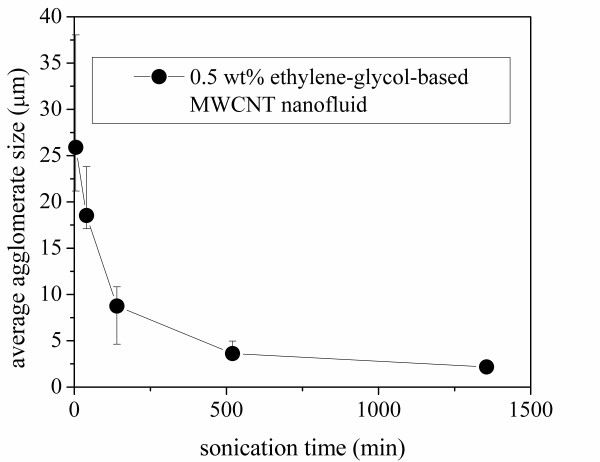
**Average agglomerate size of MWCNT-ethylene glycol nanofluids as a function of sonication time**.

In order to understand sonication time effects on the thermal conductivity and viscosity of the MWCNT-ethylene glycol nanofluid, microscopy was employed to examine the agglomerate size. Images of 6 μl droplets of nanofluids held between two glass slides are shown in Figure 9; the droplets were subjected to sonication times of 5, 40, 140, 520, and 1,355 min. With an increased sonication time, the agglomerate size becomes smaller, and the small agglomerates spread more widely with base fluid between glass slides. Micrographs of these droplets, as shown in Figure 10, confirm this statement. The magnification from the optical microscope is the same for all images as shown in Figure 10, and the scale bars correspond to a length of 200 μm. Images were recorded at four locations for each nanofluid sample (note that the edges of bubbles are shown in Figure 10a,b). The micrographs were analyzed using standard image processing, and the average agglomerate sizes were obtained for the samples. The average agglomerate size for the MWCNT-ethylene glycol nanofluids is shown as a function of sonication time in Figure 11. From Figures 10 and 11, it is clear that the agglomerate size in the nanofluids decreased with a longer sonication time. Smaller agglomerates imply a more uniform dispersion of the nanoparticles, and this more uniform distribution probably contributes to the increase in thermal conductivity with sonication time shown in Figures 4 and 5.

In addition to changes in agglomerate size, it is also apparent from Figure 10 that the morphology of the agglomerates varied with sonication time. In the as-received condition, the MWCNTs have a large aspect ratio (length/diameter), and they were highly entangled (see Figure 1). Upon dispersion in the base fluid and sonication, the agglomerated MWCNTs appear to go through a two-stage morphological change. In the first stage, sonication appears to loosen the agglomerations, without much impact on the length of the MWCNTs: Figure 10a,b,c suggest this loosening, as progressively longer sonication time result in 'fluffier' agglomerations, with the appearance of fragmented MWCNTs in Figure 10c. With lengthier sonication times, a second stage ensues, and the entangled MWCNTs begin to break: Figure 10c,d,e shows this process. The viscosity behavior supports this description of a two-phase process. A loosening of the agglomerations apparently results in an increase in viscosity; however, once the second stage is entered and the MWCNTs begin to break up, the viscosity begins to decrease (see Figure 7). These findings are unlikely to be quantitatively general for other base fluids; i.e., there is no reason to expect that the increase in conductivity and the maximum increase in viscosity will be quantitatively the same for other base fluids. However, the trends manifested by the ethylene glycol-based MWCNT nanofluid are anticipated with other base fluids because the two-stage process of loosening agglomerations and then breaking up of the MWCNTs is expected with sonication in other fluids. Therefore, an increasing thermal conductivity and an increasing then decreasing viscosity with sonication time are expected. Moreover, it is expected that for a less viscous base fluid, such as water, for the duration of the first stage might be quite shorter than that of ethylene glycol.

### Length of the carbon nanotube

**Figure 12:**
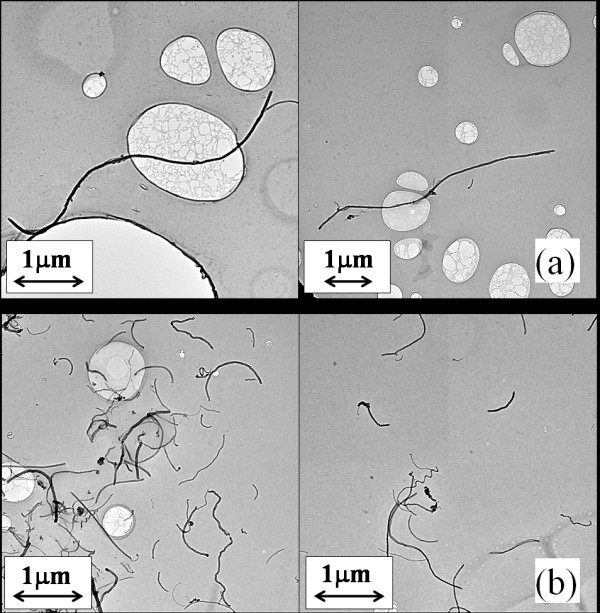
**TEM images of the MWCNT nanofluid at different sonication time: (a) 0 min, (b) 1,355 min**.

**Figure 13:**
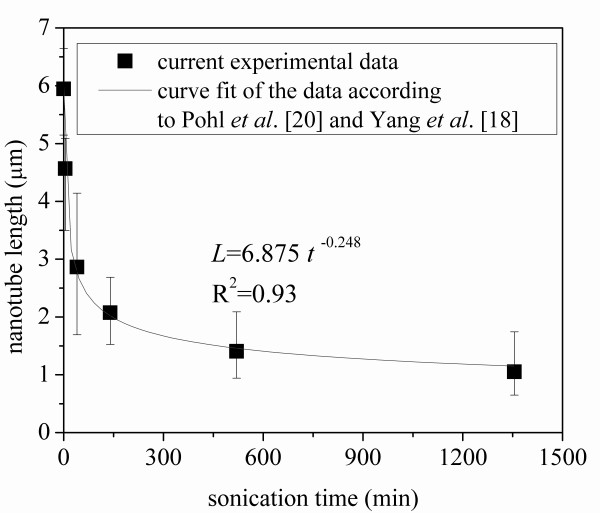
**Average length of MWCNT in nanofluids variation with sonication time**.

**Figure 14:**
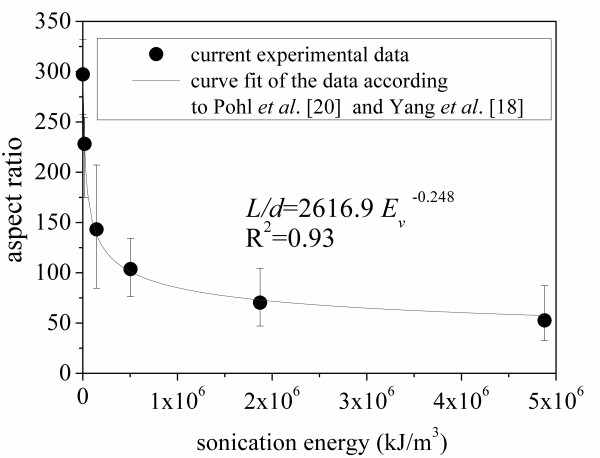
**Aspect ratio of MWCNT in nanofluids variation with sonication energy**.

Micrographs obtained from a TEM of the MWCNTs before sonication and after sonication for 1,355 min are shown in Figure 12a,b, respectively. At least five images at different locations were recorded and analyzed for each sonication time. Using standard image analysis methods, the average length of the MWCNTs was determined for each of the five images, and these values were averaged to obtain an overall average for each sonication time. Each subfigure in Figure 12 presents two representative images from two different locations. The results are presented in Figure 13, as the average length of MWCNT in ethylene glycol-based nanofluids plotted against the sonication time. The average aspect ratio of MWCNT plotted against the sonication energy input is presented in Figure 14. The aspect ratio of the carbon nanotube was calculated by dividing the length by the mean diameter of the nanotubes (20 nm). As shown in Figures 12, 13,14, as the agglomerate size is reduced, the length of the carbon nanotube is also reduced. According to Pohl et al. [20], the length of the carbon nanotube can be expressed as a function of the sonication specific energy *E*_v _(sonication energy per unit volume):

(1) $$L=\text{A}{E_{\text{v}}}^{\text{m}}$$

where *L *is the length of the carbon nanotube, and A and m are constants. Yang et al. [18] recommended an alternate form of (Equation 1), when the specific energy input is stable and the volume of dispersion is constant:

(2) $$L=\text{B}t^{\text{n}}$$

where B and n are constants, and *t *is the sonication time.

The data in Figures 13 and 14 were fit to curves of the forms given by (Equations 1 and 2). The values of the constants and the coefficients of determination, *R*^2^, are shown in the figures. The value of *n *found by Yang et al. [18] was -0.2742, which differs by 9.6% from that found in the current work; however, this is very likely a base-fluid effect. Yang et al. [18] employed oil as their base fluid. It should also be noted that Yang et al. [18] reported a decrease in thermal conductivity due to a decrease in carbon nanotube length. However, in the current work, the thermal conductivity increased with an increased sonication time. In the current work and in the work of Yang et al. [18], the as-received nanotubes had aspect ratios of about 300. However, due to the different base fluids, the changes in aspect ratio with sonication energy were different. Yang and co-workers observed a sharp decrease in the aspect ratio from 300 to 50 with a sonication specific energy input of 2.4 × 10^5 ^kJ/m^3^. They also observed an aspect ratio of less than 50 with a sonication specific energy input of more than 2.4 × 10^5 ^kJ/m^3^. In contrast, the current data show that with a sonication specific energy input of 2.4 × 10^5 ^kJ/m^3^, the aspect ratio decreased from 300 to 215. Hence, in the current work, the aspect ratio was much larger than that found by Yang and co-workers, after sonication with the same specific energy input. Even when the energy input has reached 5 × 10^6 ^kJ/m^3^, the aspect ratio was larger than 50 in the current work. Apparently, in the current work, the effect of a decrease in aspect ratio on thermal conductivity is not strong compared to the thermal conductivity increase induced by reduction of agglomerate size. Moreover, Figures 13 and 14 suggest that further sonication treatment (with a sonication specific energy input exceeding 5 × 10^6 ^kJ/m^3^) may not decrease the aspect ratio significantly, because the aspect ratio asymptotically approaches a value near 50 with energy input.

### Property relations

**Figure 15:**
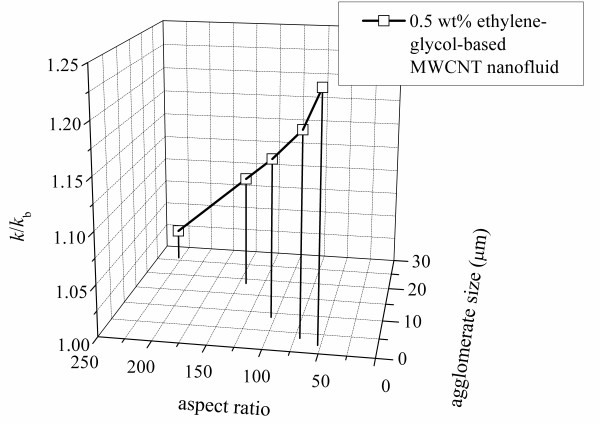
**Thermal conductivity ratio variation with nanotube aspect ratio and agglomerate size**.

**Figure 16:**
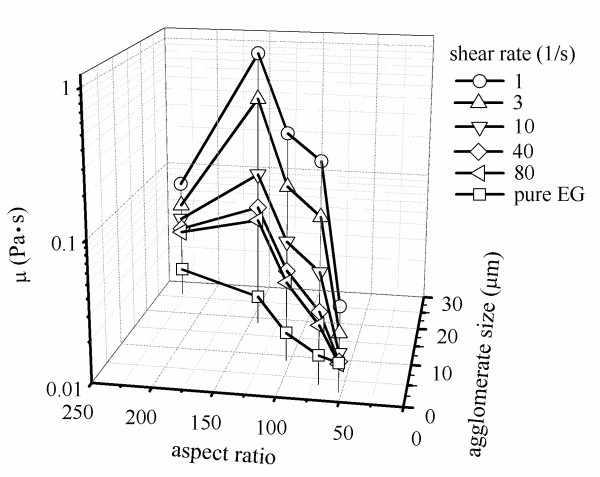
**Viscosity variation with nanotube aspect ratio and agglomerate size at different shear rates**.

The data on thermal conductivity and viscosity can be related to agglomerate size and aspect ratio, and such a model may have value in the application of ethylene glycol-based MWCNT nanofluids. In using such curve fits, caution should be maintained to recognize the limitations of the data upon which the curve fits are based: the nanofluids used a 0.25 wt% of gum arabic, and the MWCNT concentration was fixed at 0.5 wt%. Relations between the nanofluid thermal conductivity and viscosity and the MWCNT shape (nanotube aspect ratio and agglomerate size) are presented in Figures 15 and 16, respectively. Figure 15 shows the thermal conductivity plotted against the nanotube aspect ratio and agglomerate size at different sonication times. A curve fit correlation to the data of Figure 15 is shown as (Equation 3); the fit has an average deviation of 1.7% and the maximum deviation of 3.3% at the large nanotube aspect ratio and agglomerate size:

(3) $$k/k_{\text{b}}=1.236{\left(\frac{{\left(L/d\right)}^{0.2}}{D_{\text{agg}}}\right)}^{0.069}$$

where *D*_agg _is the agglomerate size, with the units of micrometer. The viscosity is plotted against the nanotube aspect ratio and the agglomerate size at different shear rates in Figure 16. The viscosity reached its maximum at a nanotube aspect ratio of 143.2 and an agglomerate size of 18.5.

## Conclusions

The sonication effects in the preparation of ethylene glycol-based MWCNT nanofluids were investigated both macroscopically and microscopically. In particular, sonication time/energy effects on thermal conductivity and viscosity of MWCNT nanofluids were explored. The thermal conductivity of the nanofluids increased nonlinearly with an increase in sonication specific energy input. The ethylene glycol-based MWCNT suspension behaved as a non-Newtonian fluid. With an increased shear rate, the viscosity of the nanofluid decreased. However, at a fixed shear rate, the viscosity of the nanofluid increased and then decreased with sonication specific energy input, and the maximum viscosity occurred at a sonication specific energy input of about 1.44 × 10^5 ^kJ/m^3^. Using microscopy and standard image analysis tools, the MWCNT agglomeration size and aspect ratio were quantified. Images were analyzed, and the results were used to explain the thermal and rheological behavior of the nanofluids. The sonication cannot only break the agglomerates, but also reduce the aspect ratio of carbon nanotubes. In the current experimental range, the thermal conductivity increases with sonication time/energy because the effect on breaking agglomerates was more significant than the effects related to reducing the MWCNT lengths. However, the viscosity of nanofluid increased during the first 40 min of sonication because agglomerates were loosened before they were broken. Thereafter, agglomerates were broken, and the viscosity of nanofluids decreased with time until it approached that of the base fluid.

## Competing interests

The authors declare that they have no competing interests.

## Authors' contributions

BR designed and carried out the experimental studies and drafted the manuscript. AMJ guide the experimental studies and revised the manuscript. All authors read and approved the final manuscript.
